# Expanded Phenotypic Spectrum of Cerebral Dysgenesis, Neuropathy, Ichthyosis, and Keratoderma (CEDNIK) Syndrome: A Rare Case Featuring Supraventricular Tachycardia and Tethered Spinal Cord

**DOI:** 10.7759/cureus.86633

**Published:** 2025-06-23

**Authors:** Noor Fuad, Raafat Hamad Seroor H Jadah

**Affiliations:** 1 Medicine and Surgery, Bahrain Defence Force Hospital, Riffa, BHR; 2 Pediatric Neurology, Bahrain Defence Force Hospital, Riffa, BHR

**Keywords:** cednik syndrome, global developmental delay (gdd), phenotypic variability, supraventricular tachycardia (svt), tethered spinal cord

## Abstract

Cerebral dysgenesis, neuropathy, ichthyosis, and keratoderma (CEDNIK) syndrome is a rare, autosomal recessive neurocutaneous disorder. It represents a progressive neurodegenerative condition caused by mutations in the synaptosome-associated protein 29 (SNAP29) gene, which encodes a member of the soluble N-ethylmaleimide-sensitive factor attachment protein receptor (SNARE) family. This protein plays a critical role in intracellular membrane fusion and protein trafficking. Mutations in SNAP29 disrupt normal cellular processes, resulting in a broad spectrum of clinical manifestations, including facial dysmorphisms, microcephaly, severe developmental delay, hypotonia, ichthyosis, and peripheral neuropathy. In this report, we describe a rare case of CEDNIK syndrome featuring novel clinical findings, supraventricular tachycardia (SVT) and a tethered spinal cord, both of which have not been previously documented in association with this syndrome. These observations contribute to the expanding phenotypic spectrum of CEDNIK syndrome.

## Introduction

Cerebral dysgenesis, neuropathy, ichthyosis, and keratoderma (CEDNIK) syndrome is a rare, autosomal recessive neurodegenerative disorder caused by mutations in the synaptosome-associated protein 29 (SNAP29) gene, located on chromosome 22 and encoding a member of the soluble N-ethylmaleimide-sensitive factor attachment protein receptor (SNARE) protein family. To date, 25 cases from 19 families have been reported worldwide [1].

SNAP29 is a soluble N-ethylmaleimide-sensitive factor attachment protein involved in vesicle fusion. In the skin, lamellar granules, located in the upper layers of the epidermis and responsible for the proper transport of lipids, proteases, and their inhibitors to the stratum corneum, undergo abnormal maturation when SNAP29 expression is diminished. This results in retention of granules, hyperkeratosis, and impaired barrier formation due to the accumulation of glucosylceramide and kallikrein-containing granules in the stratum corneum in patients with CEDNIK syndrome [2].

CEDNIK syndrome is characterized by neurological abnormalities, including global developmental delay, hypotonia, and peripheral neuropathy, along with dermatological manifestations such as ichthyosis and keratoderma. Common magnetic resonance imaging (MRI) findings include hypoplasia or dysplasia of the corpus callosum and polymicrogyria (PMG) [3].

In certain cases, complications related to neurological and skin manifestations may lead to a reduced life expectancy. The general prognosis for CEDNIK syndrome is unfavorable [3]. In this report, we present a case that broadens the clinical spectrum by including supraventricular tachycardia (SVT) and tethered spinal cord as additional phenotypic features.

## Case presentation

A five-year-old female patient was referred to our pediatric neurology clinic for evaluation of global developmental delay and hypotonia, symptoms which became apparent at the age of one year. She was born at term via cesarean section with Apgar scores of 7 and 9 at one and five minutes, respectively, and her birth weight was 3.36 kg. Pregnancy was uncomplicated, and prenatal ultrasounds were normal.

The patient is the third child of consanguineous parents who are third cousins. Notably, one of her siblings has a confirmed diagnosis of CEDNIK syndrome, while the other two siblings are healthy.

Shortly after birth, the patient developed a brief episode of cyanosis and was admitted to the neonatal intermediate care unit (NICU) for evaluation and management of suspected neonatal sepsis. She exhibited bradycardia during the early neonatal period, which improved with stimulation and supplemental oxygen. However, approximately two hours after birth, electrocardiography (ECG) revealed supraventricular tachycardia (SVT) with a heart rate of 280 beats per minute. Management included five escalating doses of intravenous adenosine (0.05-0.25 mg/kg) and restored normal sinus rhythm.

After pediatric cardiology consultation, the patient was started on oral propranolol at 1 mg every six hours for six months, which stabilized the heart rate and rhythm, with no recurrence of SVT episodes. Transthoracic echocardiography initially revealed a 3 mm patent ductus arteriosus (PDA) along with a patent foramen ovale (PFO). A follow-up echocardiographic assessment conducted one year later demonstrated spontaneous closure of both defects, with no residual abnormal findings.

**Figure 1:**
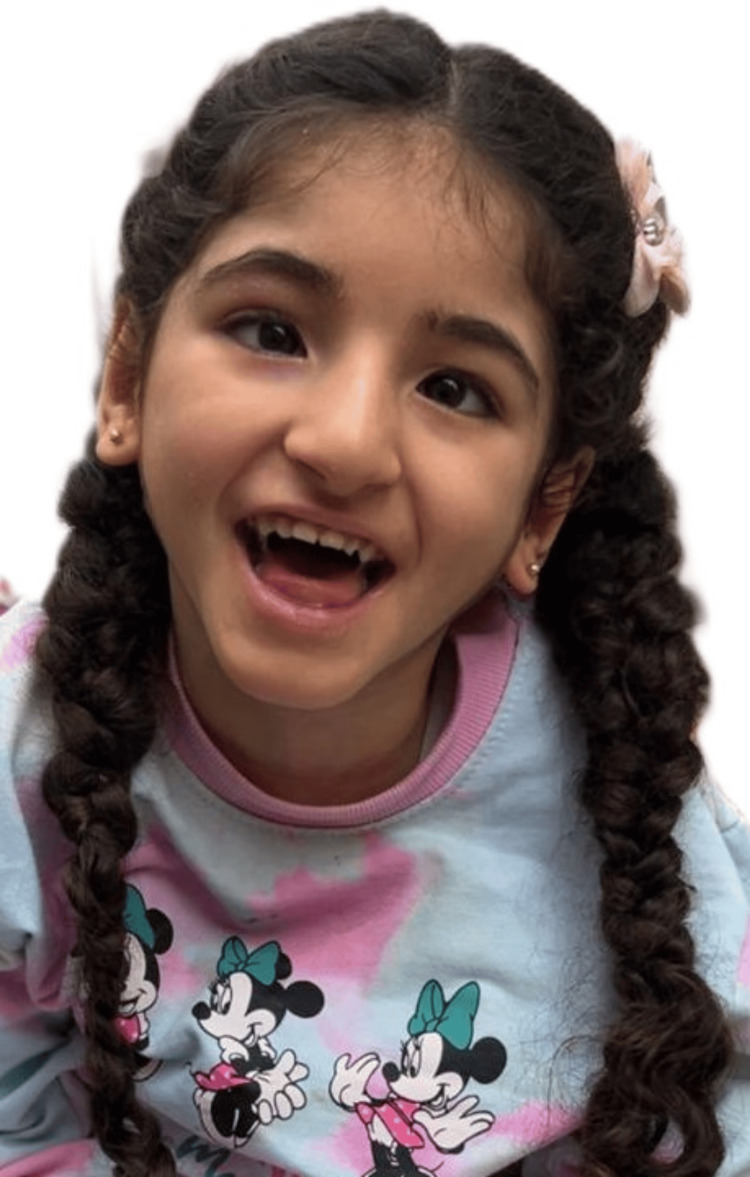
Facial dysmorphic features observed in the patient, including mild hypertelorism, strabismus, micrognathia, a peaked nose, flat forehead, and mildly low-set ears. Informed consent was obtained from the patient's legal guardians for the publication of this image.

Initial physical examination showed a head circumference of 33 cm. General inspection revealed bilateral nystagmus, left eye strabismus, with dysmorphic features in the form of micrognathia, low-set ears, mild hypertelorism, strabismus, peaked nose, and flattened forehead (Figure 1). While examining the back, a sacral indentation with a surrounding hypopigmented macule was noted. Neurological examination demonstrated generalized hypotonia, deep tendon reflexes graded +2, and motor strength of 4/5 in both upper and lower limbs. The remainder of the systemic examination was unremarkable.

Given the presence of global developmental delay, hypotonia, abnormal ocular findings, and a positive family history of CEDNIK syndrome, a comprehensive diagnostic workup was initiated.

**Figure 2:**
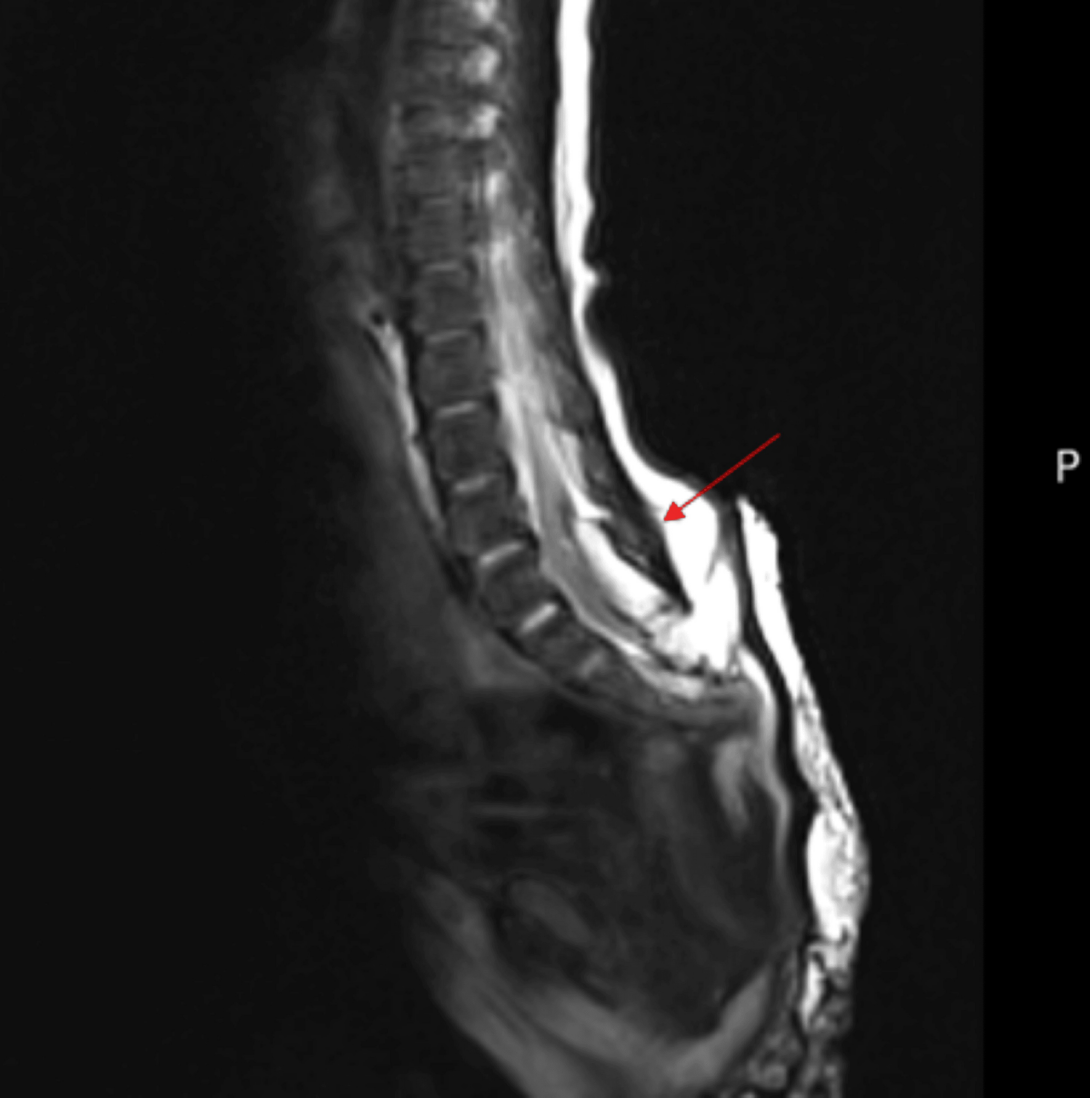
T2 sagittal view of the spine showing tethering of the spinal cord (arrow).

**Figure 3:**
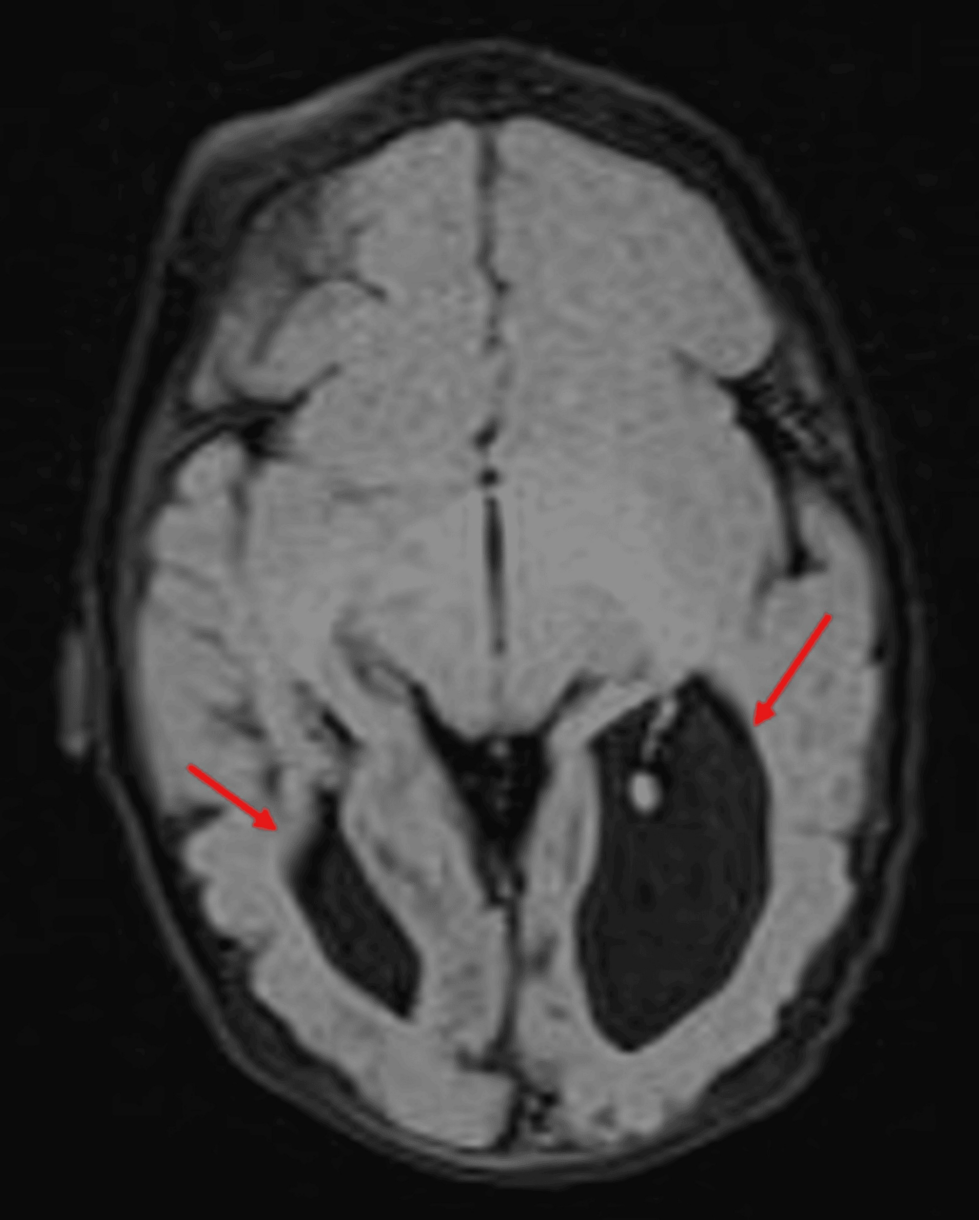
T2 FLAIR axial view brain MRI showing bilateral cerebral dysgenesis at the occipital head region (arrows). FLAIR: fluid-attenuated inversion recovery, MRI: magnetic resonance imaging

**Figure 4:**
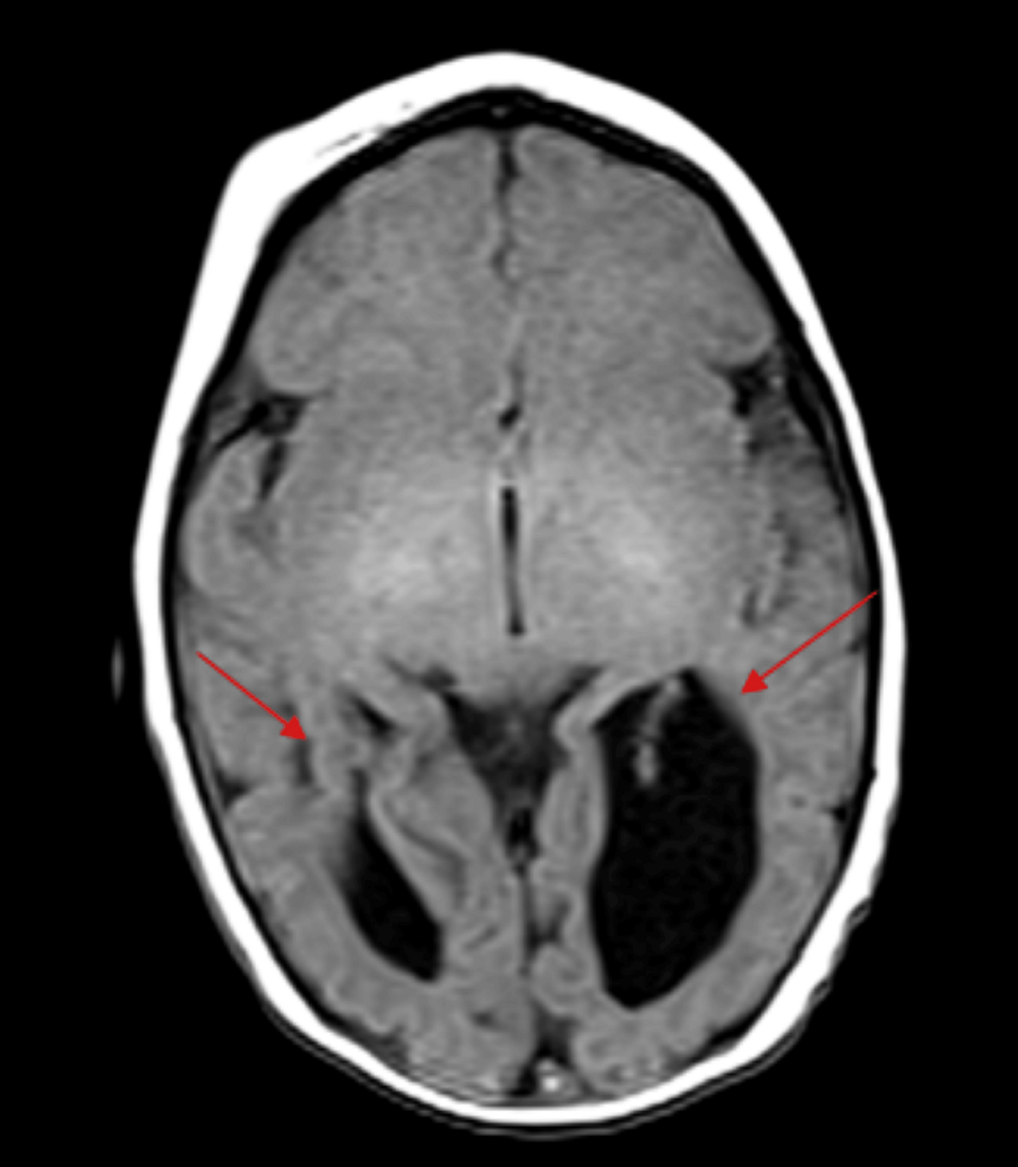
T1 axial view brain MRI showing bilateral cerebral dysgenesis at the occipital head region (arrows). MRI: magnetic resonance imaging

Neuroimaging was undertaken to evaluate for structural abnormalities, including a spinal magnetic resonance imaging (MRI) that revealed a tethered cord (Figure 2). A brain MRI revealed significantly dilated occipital horns of lateral ventricles, prominent temporal horns, and partial dysgenesis of the corpus callosum with reduced size of body and near-complete absence of isthmus (Figures 3, 4).

Based on the clinical presentation and patient history, whole exome sequencing (WES) was performed, which identified a pathogenic variant c.487dup p.(Ser163Lysfs*6) in apparent homozygosity in the SNAP29 gene, thereby confirming the diagnosis of CEDNIK syndrome. The full genetic sequencing results are available in Table 1.

**Table 1:** Whole exome sequencing confirming homozygous pathogenic variant c.487dup p.(Ser163Lysfs*6) in the SNAP29 gene. CEDNIK: cerebral dysgenesis, neuropathy, ichthyosis, and keratoderma

Section	Details
Result	The pathogenic variant c.487dup p.(Ser163Lysfs*6) was detected in apparent homozygosity in the SNAP29 gene.
Interpretation	This result indicates that the familial variant c.487dup p.(Ser163Lysfs*6) was detected in apparent homozygosity in the SNAP29 gene and confirms a genetic etiology for the clinical presentation of this patient. The c.487dup p.(Ser163Lysfs*6) variant in the SNAP29 gene is described in the literature in patients with CEDNIK syndrome and polymicrogyria syndrome (PMID: 28388629, 21073448). This variant is also reported in ClinVar (ID: 50295) as a pathogenic variant, one submitter, and is present in the population database gnomAD (0.0032%), with 8 heterozygous individuals reported and 0 homozygous. Furthermore, due to its nature, a frameshift variant located in exon 3 (of 5 exons), it is predicted to create a premature stop codon and therefore to produce a truncated protein and/or its decreased expression by mRNA degradation. The brother's result and the obtained result suggest the segregation of the detected variant with the disease in the family. With the available information, it should be classified as a pathogenic variant.
Conclusion	Pathogenic variants in the SNAP29 gene cause CEDNIK syndrome (MIM 609528), with an autosomal recessive pattern of inheritance.

At five years of age, during a routine follow-up visit, the patient continued to exhibit profound global developmental delay. She remained unable to walk independently or crawl, although she was able to sit without assistance. Marked delays in speech and language development were noted, along with significant cognitive impairment and an inability to communicate effectively with her parents. The patient is fully vaccinated in accordance with the national immunization schedule of the Kingdom of Bahrain.

## Discussion

CEDNIK syndrome is a rare neurocutaneous disease first described by Sprecher et al. in 2005 [4]. It is an autosomal recessive disease that results from decreased expression of SNAP29 protein mapped to the 22q11.2 gene, a SNARE protein that plays an important role in vesicle fusion [5].

Thus, loss-of-function SNAP29 results in a disease that exhibits a mixture of neurological and dermatological symptoms. Neurologically affected people frequently have global developmental delays, hypotonia, microcephaly, and brain deformities such as corpus callosum dysgenesis and cortical dysplasia. Dermatological symptoms include congenital ichthyosis (dry, scaly skin) and palmoplantar keratoderma (thickness of the skin on the palms and soles) [6].

In previously reported cases of CEDNIK syndrome involving 19 patients compared with six new cases, our patient shares several hallmark features, such as global developmental delay and cerebral dysgenesis. Notably, while seizures were reported in approximately 36.8% of previous patients (7/19) and 50% (3/6), our patient had no history of seizures, which may suggest phenotypic variability. In contrast, features such as strabismus were observed in 67% of patients in the newer cohort (4/6) but only 10.5% in the earlier series (2/19), further supporting its emerging association with SNAP29 mutations. Similarly, hypomyelination was observed in 66.7% of recent cases (4/6) versus only 15.8% previously (3/19), and constipation, which was not reported in earlier patients, was present in 50% of the new cases (3/6). Reports of early puberty in pubescent patients were comparable across groups (66.7% of new cases versus 50%) [3]. Additionally, our case exhibited SVT and a tethered spinal cord that were absent from earlier reports. These findings expand the phenotypic spectrum of CEDNIK syndrome and suggest a new association of SVT and tethered spinal cord in future patients diagnosed with CEDNIK syndrome.

The standard diagnosis of CEDNIK syndrome is made through whole exome genetic testing, with the presence of neuropathy, keratoderma, and ichthyosis serving as important diagnostic clues [7]. Our patient underwent molecular genetic testing in the form of whole exome sequencing (WES), which confirmed a genetic mutation in SNAP29, consistent with a diagnosis of CEDNIK syndrome.

While cerebral dysgenesis and peripheral neuropathy are well-described features, supraventricular tachycardia (SVT) and tethered spinal cord are not typically associated with CEDNIK syndrome. Our case is unique in highlighting tethered spinal cord and SVT as potential new phenotypic features.

## Conclusions

CEDNIK syndrome is a rare genetic disorder characterized by neurodevelopmental delay, ichthyosis, and keratoderma. Diagnosis is based on clinical assessment supported by neuroimaging, with confirmation typically requiring whole exome sequencing. The prognosis for patients with CEDNIK syndrome is generally poor.

With the increasing number of reported cases, the clinical spectrum of CEDNIK syndrome continues to expand. Identifying new phenotypic features may facilitate earlier diagnosis, highlight the importance of early genetic counseling, especially in families with a history of affected children, and guide the development of supportive management strategies to improve outcomes. A newly observed association, such as supraventricular tachycardia and tethered spinal cord, should be considered part of the phenotypic spectrum in patients diagnosed with CEDNIK syndrome.
